# The epidemiology of inflammatory bowel disease: Clues to pathogenesis?

**DOI:** 10.3389/fped.2022.1103713

**Published:** 2023-01-17

**Authors:** Stephen M. Borowitz

**Affiliations:** Division of Pediatric Gastroenterology, Hepatology and Nutrition, University of Virginia, Charlottesville, VA, United States

**Keywords:** inflammatory bowel disease, Crohn's disease, ulcerative colitis, epidemiology, genetics, environment, etiology, pathogenesis

## Abstract

Historically, inflammatory bowel disease (IBD) was most common in North America and Europe and more common with a north-south gradient. Over the past century, there has been a marked increase in IBD in general and in childhood IBD in particular and over the past 50 years IBD has spread into the developing world. The greatest risk factor of developing IBD is an affected family member. Concordance rates between dizygotic twins is ∼4% and ∼50% in monozygotic twins, and more than half of pairs are diagnosed within 2 years of each other. Nevertheless, most patients with IBD do not have an affected family member. More than 200 genes are associated with an increased risk for IBD, but most associations are weak with odds ratios between 1.2 and 2.0 suggesting the environment plays a role. IBD is more common in urban than rural regions and is associated with “good standards” of domestic hygiene during childhood. People who migrate from areas with a low incidence to areas with a high incidence of IBD have an increased risk of developing IBD and the younger they are when they migrate, the greater their risk of developing IBD. Moreover, people who migrate from regions with a high incidence to areas with a low incidence of IBD have a decreased risk of developing IBD. Together, these findings strongly suggest particular environmental exposures occurring early in life may trigger inflammatory bowel disease in genetically susceptible individuals. The key is figuring out what those exposures might be.

Inflammatory bowel disease (IBD), comprised of ulcerative colitis, Crohn's disease and IBD-unclassified, is a chronic inflammatory disorder of the gastrointestinal tract. While the etiology of IBD remains uncertain, the most widely accepted theory is that the disease is caused by an aberrant or dysregulated immune response directed at the intestinal microbiota in genetically susceptible individuals and it seems fairly clear that individuals suffering from IBD have disordered or disrupted intestinal microbiota however it remains unclear whether this dysbiosis is the cause or the result of the disease (1, 2).

While IBD is predominantly a disease of young adults, it can occur at any age and approximately 25% of patients with IBD will present before 20 years of age. The peak incidence of IBD in childhood is during adolescence, however approximately 20% of children with IBD will present before 10 years of age, and approximately 5% will present before 5 years of age (3). The incidence of IBD in general, and childhood onset IBD more specifically, is increasing throughout much of the world, and a number of recent studies have found rapid growth of very early onset IBD in some but not all of those regions where there have been long-standing high rates of both pediatric and adult onset IBD (4, 5).

IBD was first recognized as a distinct entity in Western Europe and North America towards the end of the eighteenth century around the dawn of the industrial revolution and by the early 1900's ulcerative colitis had become a well-recognized disorder. In 1932 Crohn and colleagues published their landmark paper describing regional ileitis and differentiating it from ulcerative colitis (6). Throughout the second half of the twentieth century the incidence of both Crohn's disease and ulcerative colitis rapidly accelerated throughout industrialized areas of Western Europe and North America (7, 8).

Up until fairly recently, IBD was almost exclusively recognized in industrialized areas of Western and Northern Europe, North America, Australia and New Zealand and throughout much of the 20th century, these regions experienced a dramatic increase in the incidence of IBD. Most recent data suggest the incidence of IBD has stabilized in these highly endemic areas, however the incidence of childhood IBD continues to rise (2, 4). Over the past 50 years, these traditional geographic boundaries have largely disappeared and IBD has now become a global disease (7–9). Since the end of the 20th century, newly industrialized and developing countries in Asia, Latin America, South America and Africa have seen dramatic rises in the incidence of IBD (2, 4). In areas with a historically low incidence of IBD, as the incidence of IBD increases, ulcerative colitis tends to emerge first followed by Crohn's disease and over time Crohn's disease comes to predominate (4). This same pattern parallels what happened in North America and Northern Europe during the last century (4).

The single greatest known risk factor for developing IBD is having a close family member with IBD and there appears to be a greater genetic association for childhood onset than adult onset IBD and the younger the child at the time of diagnosis, the greater the association (10, 11). The familial nature of IBD was recognized more than 100 years ago, and since then, numerous studies have reported family clustering of the disease. It is estimated that between 5% and 23% of people with IBD have a first-degree relative with IBD (12) and among families with multiple affected family members, there tends to be a fairly high degree of clinical similarity among affected family members for disease location, disease behavior, age at the time of diagnosis and the presence and character of extra-intestinal manifestations of the disease (13).

In most studies, the risk of developing IBD appears to be greatest if you have an affected mother or an affected sibling (14, 15). If you have a sibling who has been diagnosed with Crohn's disease, your relative risk of developing IBD has been estimated to be between 13 and 36 and your relative risk of developing IBD is between 7 and 17 if you have a sibling diagnosed with ulcerative colitis (15). 1.7% of siblings will be diagnosed within 10 years of one of their siblings being diagnosed with IBD, and the estimated 10 year cumulative incidence rate for IBD is 0.5%–3.5% in first degree relatives. Moreover, the risk of developing the disease increases as the number of family members affected with IBD increases and this association appears to be greatest for pediatric onset Crohn's disease (10). The risk of IBD in offspring increases dramatically when both parents suffer from IBD; case series have estimated the risk of IBD in offspring to be 33%–52% depending on the duration of follow up (13, 16).

In studies of twins, concordance rates for dizygotic pairs has been reported between 3% and 5% whereas concordance rates for monozygotic twins has been reported between 20% and 60% with higher rates among pairs with Crohn's disease than pairs with ulcerative colitis, and among pairs with Crohn's disease, the majority have disease in the same location of the bowel (16, 17). In a recent long-term study of 91 twin pairs from the UK in whom at least one twin had IBD, 42 pairs had one or both twins with Crohn's disease and 41 pairs had one or both twins with ulcerative colitis. The concordance rate for Crohn's disease was 65% among monozygotic twins and 9.1% for dizygotic twins and the concordance rate for ulcerative colitis was 17.6% for monozygotic twins and 9.4% for dizygotic twins. For concordant pairs, the median time between the diagnosis of the first and second twin was 4 years; 3 years for monozygotic pairs and 13 years for dizygotic pairs. Among twins with ulcerative colitis, the median time between diagnosis of the first and second twin was 4 years for monozygotic pairs and 15 years in the dizygotic pairs (17).

Genome-wide linkage analyses have identified more than 200 genes that are associated with an increased risk of developing IBD although most of the associations are relative weak with odds rations generally in the range of 1.2–2 (18). Many of these genes are also associated with other autoimmune disorders including type 1 diabetes, ankylosing spondylitis, psoriasis, primary immunodeficiencies, Mendelian susceptibility to mycobacterial disease, and leprosy. These genes are involved in an array of immune functions including microbial recognition, innate immunity, lymphocyte activation and proliferation, cytokine production, and intestinal barrier function (18).

Among Caucasians, loss of function mutations in the NOD2/CARD15 gene can be identified in between 10% and 30% of patients with Crohn's disease (19). NOD2/CARD15 is expressed in epithelial Paneth cells, neutrophils, and macrophages, and recognizes bacterial cell wall peptidoglycan. NOD2/CARD15 is a member of a group of cytoplasmic pattern recognition receptors expressed in many different cell types that induces the NF-kB pathway when NOD2/CARD15 recognizes muramyl dipeptide from bacterial peptidoglycan (20). Roughly 10% of healthy Caucasians carry NOD2/CARD15 variants associated with Crohn's disease however the majority of these people do not develop IBD; carrying a mutation in NOD2/CARD15 confers a three-fold risk of developing Crohn's disease among heterozygotes and a 20-fold increased risk for homozygotes with approximately 5% penetrance (19).

Gene wide linkage analyses can at most explain roughly a quarter of heritability in IBD (21) and while concordance rates among monozygotic twins is high, in none of the published studies does it approach 100% indicating genes aren't enough to cause IBD and the environment and/or epigenetic factors must be playing a role. Furthermore, the rapid rise in the incidence of IBD and its geographic spread over the past 50 years has occurred much too fast to be explained by changes in our genetic make-up.

A role for environmental factors in the pathogenesis of IBD is strongly suggested by the geographic pattern of the disease. IBD first emerged in the northernmost parts of Europe and North America and over the subsequent hundred years it has migrated southward (4) and while this distinct north-south gradient appears to persist in the northern hemisphere, it seems to have diminished over time. While data are limited, there also appears to be a south-north gradient in the southern hemisphere with some of the highest incidence rates in the world in Australia and New Zealand (2, 22). Moreover, a number of studies have demonstrated markedly different incidence rates of IBD within different regions of a number of countries (2).

Studies among immigrant populations further implicate the importance of the environment in the etiology of IBD (2, 23–26). A number of studies have found that when people migrate from an area with a low incidence of IBD to an area with a high incidence of IBD, their risk of developing IBD increases significantly and the younger they are when they migrate to the area of high incidence, the greater their risk is of developing IBD (2, 23, 24). Moreover, people who migrate from regions with a high incidence of IBD to areas with a low incidence of IBD tend to have a decreased risk of developing IBD and the younger they are when they migrate to the area of low incidence, the lower their risk is of developing IBD (2, 25). However, these findings are not universal. While people who immigrate from South Asia to Canada and England have an increased risk of developing IBD, this is not true of people who immigrate to Canada from China suggesting that while environmental exposures play a role in the pathogenesis of IBD, there may be factors that mitigate or at least attenuate the risk of developing IBD despite those exposures (26).

As IBD has become increasingly more common in areas where the incidence has historically been very low, it has become increasingly clear that the incidence of IBD appears to rise with the transition from “developing” to “developed” indicating there is linkage between IBD and lifestyle and the environment (2). In nearly all studies that have looked at it, IBD is more common in urban as compared to rural regions (27) and there appears to be a positive correlation between socioeconomic status and the likelihood of developing IBD (2). A number of environmental factors associated with urbanization and industrialization have been proposed to play a role in the pathogenesis of IBD including exposure to antibiotics during childhood as well as exposure to dietary emulsifiers both of which may produce dysbiosis (2, 8, 28). It is long been proposed that the dietary changes associated with urbanization and industrialization play a role in the pathogenesis of IBD (26). Diet clearly influences the composition of the intestinal microbiome and a Western diet with large amounts of red meat, high fat foods, and refined sugars has been linked to intestinal inflammation (29). Moreover, a number of studies have demonstrated that ingestion of a liquid formula diet as the only source of nutrition for a period of time improves both symptoms and intestinal inflammation in people suffering from Crohn's disease (30), however these positive results are not universal (31). Despite these observations and animal studies that have demonstrated a role for diet in the maintenance and regulation of intestinal barrier function, the observational studies that have investigated the role of diet in the pathogenesis of IBD have yielded inconclusive and at times contradictory results (29).

It has also been proposed that this association between higher socioeconomic status and IBD is due to the hygiene hypothesis which proposes that decreased infectious diseases, diminished parasitic infestations, the greater use of vaccinations and of antibiotics particularly early in life and general improvements in food, water and housing sanitary conditions increases the risk of developing auto-immune and chronic inflammatory diseases (14, 32). Limited exposure to a number of common infections and environmental antigens during early childhood may lead to an increased susceptibility to an aberrant immune response to various infectious and/or other environmental exposures in genetically susceptible individuals (27). There are a number of studies that have identified an inverse correlation between markers of domestic hygiene early in life and the risk of developing IBD such as living in a rural region, exposure to farm animals, increased family size, bedroom sharing, urbanization and pet ownership (33, 34). The hygiene hypothesis may also explain the inverse relationship between Helicobacter pylori infection and IBD (35). In some studies, the strongest and most consistent predictor of developing IBD is having a mother with IBD. While this could reflect genetic effects, it also likely reflects environmental effects early in life as young children typically share the environment very closely with their mothers and studies have shown that the gut microbiome of young children closely mirrors the microbiome of their parents not only related to living conditions but also to a shared diet (14).

As discussed above, most of the epidemiologic data suggest IBD is more common in urban settings and among people of higher socioeconomic status and with “greater” domestic hygiene particularly during childhood however there are numerous intriguing reports of clustering of cases of IBD in rural agricultural settings. Comes described ten married couples with IBD in Nord Pas de Calais France and Liege county Belgium. In nine of the ten couples, neither spouse had any symptoms of IBD before marriage, and IBD developed in both after marriage (36). Bennett identified 21 couples both of whom had IBD in New York State. In 14 of the couples, the second spouse developed their disease after they were married (37). Allan and colleagues characterized 11 cases of IBD that developed in a small British community of 2,000 people over 15 years representing an incidence of 36.5/100,000 per year (38). Reilly and Robertson described Crohn's disease in four women ages 21–30, who had been close elementary and high school friends (39). Aisenberg and Janowitz reported IBD in three close college friends, two of whom developed Crohn's disease and the third developed ulcerative colitis after sharing an apartment, bathroom, and kitchen (40). Van Kruiningen reported seven cases of IBD in a high school class of 285 people that developed over 14 years in a rich agricultural area in Western Minnesota representing a prevalence of 2,400/100,000 (41). Similarly Kruiningen and Joosens described a cluster of five cases of Crohn's disease in a small agricultural village in France with a population of approximately 450. The homes of the affected people were all within 100 meters of one another and they all shared a common water source (42). In a Belgian study of 21 families comprising 159 people, 75 suffered from IBD, 74 of whom suffered from Crohn's disease. The mean age at onset of symptoms was 24 and all but two of the families lived in rural regions surrounded by agricultural fields (43). More recently, we reported 15 unrelated children and teenagers between 5 and 18 years of age living in a suburban town in Central Virginia surrounding by dairy farms who were diagnosed with inflammatory bowel disease, 11 with Crohn's disease and four with ulcerative colitis. Three unrelated friends who lived in close proximity developed symptoms of IBD within 7 months of each other. None of the children were related and none of them had a family history of inflammatory bowel disease (44).

These clusters of IBD in largely rural agricultural regions point away from a purely genetic cause of IBD as well as pointing away from the hygiene hypothesis as the cause of IBD at least in some cases, and rather suggest that at least in some cases, environmental transmission of the disease may occur, perhaps transmission of a modestly contagious microbial agent with a long latent period. There have long been thoughts IBD might be the result of a chronic enteric infection given its resemblance to intestinal tuberculosis, *Yersinia enterocolitica* infection, and chronic Salmonellosis as well as its propensity to relapse and remit like tuberculosis, syphilis and Lyme disease. Furthermore, some patients with IBD respond to antibiotic therapy (1, 20, 45).

A number of microorganisms have been postulated to play a role in the pathogenesis of IBD however *my*obacterium avium subspecies paratuberculosis (MAP) may be the one that has been most intensely investigated (19). The similarities been Crohn's disease and intestinal tuberculosis were first written about in the early 1900s (46). Around the same time, it was recognized that MAP was the cause of Johne's disease which is a severe granulomatous enteritis pathologically similar to Crohn's disease that occurs predominantly in ruminants but can effect numerous other species including primates (1, 47). As discussed previously a substantial number of people suffering from Crohn's disease carry loss of function mutations in the NOD2/CARD15 gene, and in dairy cattle, polymorphisms of NOD2/CARD15 are associated with Johne's disease and NOD-2/CARD15 deficient mice have impaired resistance to mycobacterial infections (48).

Given all this information, it seems increasingly possible and even likely that IBD is not a single disease, but rather a heterogenous group of disorders with a final common pathway of inability to resolve an inciting inflammatory event (49). There is substantial evidence that perturbation of the intestinal microbiome plays a crucial role in the development and/or maintenance of IBD however it is quite possible the role the intestinal microbiome plays in this process varies in different individuals. In some people IBD may be the result of dysbiosis with the imbalance between beneficial and harmful bacteria triggering an aberrant immune response. In other individuals, IBD may be triggered by some form of disruption to the intestinal barrier triggering a defective immune response. Finally, in some individual suffering from IBD, the disease may represent persistence of a low grade pathogen that they are unable to eliminate. Together these observations strongly support the idea that environmental exposures may trigger inflammatory bowel disease in genetically susceptible individuals however the key is figuring out what that exposure or exposures those might be.
